# Immunoprevention approaches for cancer prevention

**DOI:** 10.1186/2051-1426-2-S1-P7

**Published:** 2014-02-24

**Authors:** Asad Umar

**Affiliations:** 1 Gastrointestinal & Other Cancers Research Group, Division of Cancer Prevention, NCI, Rockville, Maryland, USA

## 

Here I propose current and future strategies to develop immunoprevention approaches for cancer prevention. A major challenge in the development of effective immunopreventive agents is the recognized requirement for the simultaneous co-development of multiple agents. Infectious agents are linked to approximately 15% to 20% of all cancers worldwide. Viruses are the infectious agents most commonly associated with cancer causation but bacteria and parasites may also have a carcinogenic effect. Oncoviruses include human papillomavirus (cervical and other cancers), Epstein-Barr virus (B-cell lymphoproliferative disease and nasopharyngeal carcinoma), Kaposi's sarcoma-associated herpesvirus (Kaposi's sarcoma and primary effusion lymphomas), hepatitis B and hepatitis C viruses (hepatocellular carcinoma), and human T-lymphotropic virus-1 (adult T-cell leukemia/lymphoma). Current progress and our development of these areas include prophylactic vaccines (general naïve population to prevent infection in the first place) (e.g., HPV, HBV, HCV etc.) and therapeutic vaccines (treating already infected individuals with an intent to delay or prevent cancer onset) (e.g., HPV, EBV, HBV, HCV, and H. pylori etc.).

